# Formulation of Novel Composite (Activated Nanoclay/Hydrocolloid of *Nostoc sphaericum*) and Its Application in the Removal of Heavy Metals from Wastewater

**DOI:** 10.3390/polym14142803

**Published:** 2022-07-09

**Authors:** David Choque-Quispe, Carlos A. Ligarda-Samanez, Betsy S. Ramos-Pacheco, Aydeé M. Solano-Reynoso, Justiniano Quispe-Marcatoma, Yudith Choque-Quispe, Diego E. Peralta-Guevara, Edgar L. Martínez-Huamán, Odilon Correa-Cuba, Mery Luz Masco-Arriola, Washington Julio Lechuga-Canal, Fred Montalvo Amanca

**Affiliations:** 1 Water Analysis and Control Research Laboratory, Universidad Nacional José María Arguedas, Andahuaylas 03701, Peru; bsramos@unajma.edu.pe (B.S.R.-P.); deperalta@unajma.edu.pe (D.E.P.-G.); 2 Department of Agroindustrial Engineering, Universidad Nacional José María Arguedas, Andahuaylas 03701, Peru; caligarda@unajma.edu.pe; 3 Food Nanotechnology Research Laboratory, Universidad Nacional José María Arguedas, Andahuaylas 03701, Peru; 4 Department of Environmental Engineering, Universidad Tecnológica de los Andes, Andahuaylas 03701, Peru; ayma_21@hotmail.com; 5 Faculty of Physical Sciences, Universidad Nacional Mayor de San Marcos, Lima 15081, Peru; jquispem@unmsm.edu.pe (J.Q.-M.); fred.montalvo@unmsm.edu.pe (F.M.A.); 6 Centro de Investigaciones Tecnológicas, Biomédicas y Medioambientales, Callao 07041, Peru; 7 Department of Environmental Engineering, Universidad Nacional José María Arguedas, Andahuaylas 03701, Peru; ychoque@unajma.edu.pe; 8 Department of Education and Humanities, Universidad Nacional José María Arguedas, Andahuaylas 03701, Peru; emartinez@unajma.edu.pe; 9 Department of Basic Sciences, Universidad Nacional José María Arguedas, Andahuaylas 03701, Peru; odiloncorrea@unajma.edu.pe; 10 Department of Chemical Engineering, Universidad Nacional de San Antonio Abad del Cusco, Cusco 08000, Peru; mery.masco@unsaac.edu.pe (M.L.M.-A.); washington.lechuga@unsaac.edu.pe (W.J.L.-C.)

**Keywords:** activated nanoclay, heavy metals, hydrocolloid, adsorption isotherm, adsorption kinetics

## Abstract

The removal of heavy metals from wastewater is an environmental challenge which demands the use of environmentally friendly materials that promote a circular economy. This study aimed to apply a novel composite of an activated nanoclay/hydrocolloid in the removal of heavy metals from wastewater. A composite blended under pressure was prepared with spray-dried hydrocolloid derived from *Nostoc sphaericum* algae and activated nanoclay in an acid medium and 1M NaCl. The composite and components were analyzed through infrared (IR), X-ray (XR), ζ potential, cation exchange capacity (CEC), particle size, and SEM images. The composite was subjected to the adsorption of heavy metals (Pb, As, Zn, and Cd) at pH 4.5 and the removal percentage, kinetics, and adsorption isotherms were evaluated. It was observed that the activated nanoclay and the composite that presented a particle size of around 400 nm significantly increased (*p*-value < 0.05) the CEC, ζ potential, the functional groups, and chelating components, removing heavy metals above 99% for Pb, As 33%, Cd 15%, and Zn 10%. Adsorption kinetics was adjusted to the pseudo second-order model (*R*^2^ > 0.98), and the Langmuir and Freundlich models better represented the sorption isotherm at 20 °C. The formulated composite presents a good ability to remove heavy metals in wastewater.

## 1. Introduction

Anthropic activities cause deterioration in the environment and its resources; consumerism and the frequent renewal of materials and technology generate silent polluting residues due to their low but highly harmful concentrations, such as heavy metals [1,2,3,4].

Environmental contamination by metals has been identified as one of the most relevant environmental problems due to its harmful effects on the environment and human health, caused by its mobility in aquatic systems, its toxicity, and its high bioaccumulative capacity, reaching levels of concentration that are toxic for life [3,4], which in most cases are difficult to remove and is very expensive.

Currently, friendly technologies are being developed for water treatment, capable of reducing or eliminating the concentration of metal ions [5], these involve the use of biodegradable materials, mainly from plant sources and inert materials such as clays [2,6,7,8].

Bioadsorption is proposed as a highly efficient wastewater treatment alternative for the removal of heavy metals, due to the low costs of implementation and maintenance in terms of the mechanisms for capturing metal ions. They are very varied and depend on the metal of interest and the type of bioadsorbent material to be evaluated [9,10].

Bioadsorbents can be materials from microbial flora, algae, plants, residual biomass, and agro-industrial products [11]. One of the alternatives is to use a biopolymer, which is low cost and easy to handle compared to conventional techniques, such as activated charcoal and ion exchange resins [2,4]. There are other separation techniques, such as filtration, flocculation, electrodeposition, and precipitation; however, these methodologies generate waste sludge, treatment, and disposal of which constitutes an additional cost to the depuration process [11,12,13].

One of the more promising materials is based on the use of natural biopolymers of plant origin, specifically algae [14,15,16], due to their high content of hydrocolloids, which present chelating capacity, thus allowing the removal of heavy metals from contaminated water [10,16,17]. *Nostoc sphaericum*, commonly named Murmunta, Nostoc, Llullucha, Chusuro, and Crespito, has qualities that make it potential for use regarding these purposes.

On the other hand, the use of inert materials with a high affinity for heavy metals in aqueous solution are widely used in wastewater treatment, with clays standing out among them, due to their high adsorption and ion exchange capacity, low permeability, swelling capacity, chemical, and physical stability and high surface area [18,19,20]. This allows ionic and covalent bonds to be established between the metal cations and the surface functional groups of the clays [21,22].

These properties can improve when the clays are modified and activated in different media and treatments, and even more so if nanoclays are obtained [23,24,25,26]. Thus, nanomaterials allow optimization of adsorption processes, due to the larger contact surface and the lower amount of sorbent used.

The synergy of materials with these qualities could improve the removal potential of heavy metal cations in wastewater, which is why this study proposes the formulation of a composite activated-nanoclay/hydrocolloid of *Nostoc sphaericum* and shows the behavior of this novel material during the adsorption process.

## 2. Materials and Methods

### 2.1. Raw Material

Clay was used and collected according to the description in Table 1.

In the same way, spray-dried hydrocolloid (CH) extracted from a high Andean alga (*Nostoc sphaericum*) was used, obtained according to the methodology proposed by Choque-Quispe et al. [27].

### 2.2. Treatment and Activation of Nanoclay

The natural clay (HMB) was ground in a PM 100 planetary mill, Retsch brand (Haan, Germany), at 250 rpm for 10 min with a rotation interval of 2 min and the ground samples were sieved at 125 microns. The sieved material was treated with 10% phosphoric acid in order to remove organic matter at a ratio of 1.0 g of clay/4.0 mL of acid and stirred at 300 rpm at 60 °C for 6 h; then, it was rinsed with abundant ultrapure water, up to pH 7, and dried at 60 °C.

The clay was activated with a 1.0 M NaCl solution at a 1.0 g clay/5.0 mL ratio and stirred at 200 rpm for 24 h. Subsequently, it was rinsed with abundant ultrapure water until a conductivity of less than 10 µS/cm of the residual water was achieved and was dried at 60 °C, obtaining HMB-activated nanoclay (HMB-act) [25,28].

### 2.3. Determination of Cation Exchange Capacity

The cation exchange capacity (CEC) of natural and activated clay (HMB) was determined by the sum of the compulsive changes of Al, Ca, Mg, K, and Na, by Ba; the exchangeable cations were removed with a 0.1 mol/L BaCl_2_ solution at a 1/12 (*w*/*v*) ratio of clay/BaCl_2_ solution, which was stirred at 200 rpm for 1 h.

Then, it was centrifuged at 3000× *g* for 10 min and this operation was repeated three times. The supernatants were collected in a container, and the exchangeable ions (Al, Fe, Ca, Mg, Mn, K, and Na) were measured using an inductively-coupled plasma optical emission spectrometer, Shimadzu, model ICP-OES 9820 (Kyoto, Japan). Calibration curves were prepared for the ions under study, with a regression coefficient, *R*^2^ > 0.995. Readings were performed in axial mode with an argon gas flow of 10 L/min with 30 s plasma exposure and 30 s rinses at 60 rpm between samples.

### 2.4. Clay and Hydrocolloid Characterization 

The nanoactivated clay (HMB-act) samples and the hydrocolloid were taken to a Nicomp, nano ZLS, Z3000 (Billerica, MA, USA), in order to determine particle size and ζ potential by dynamic light scattering (DLS). According to the methodology proposed by Choque-Quispe et al. [27], microphotographs were taken through a scanning electron microscope, Thermo Fisher, model Prisma E (Waltham, MA, USA). IR analysis was determined through a Fourier transform infrared spectroscopy (FTIR), Thermo Fisher, model Nicolet IS50 (Waltham, MA, USA), in transmittance mode in the range of 4000 to 400 cm^−1^ and resolution of 4 cm^−1^. X-ray diffraction analysis was performed using a Bruker diffractometer, model D8-Focus (Karlsruhe, Germany), (Cu Kα1 = 1.54 Å) at 40 kV and 40 mA, and a PSD Lynxeye detector.

### 2.5. Composite Preparation

The hydrocolloid and the clay (natural and activated) (HMB-Act/CH) were homogeneously mixed at a ratio of 1/4 (*w*/*w*), respectively, and pressure molded, obtaining tablets (adsorbent composite).

### 2.6. Evaluation of Metal Adsorption

A multimetal solution of 10 ppm As, 10 ppm Cd, 10 ppm Pb, and 10 ppm Zn at pH 4.5 was prepared (simulating mine tailings wastewater). 50 mg of the adsorbent composite was added to 500 mL of multimetal solution and stirred at 100 rpm for 120 min, then an aliquot was taken and filtered at 0.45 µm [17]. Then, a reading using an ICP-OES 9820 Shimadzu (Kyoto, Japan) was taken, and calibrated with As, Cd, Pb, and Zn standards (Calibration solution STD, SCP Science, Baie-d’Urfé, QC, Canada), reporting regression coefficient *R*^2^ greater than 0.995 for all cases. The results of the adsorption were expressed as the percentage of removal in the residual water, through the relationship between final concentration and initial concentration of each metal.

### 2.7. Determination of Adsorption Isotherms

Adsorption isotherms show how adsorptions occur and also serve as a design parameter in treating heavy metals in wastewater [29].

An amount of 250 mL of As, Cd, Pb, and Zn multimetal solution of 10, 50, 100, 150, 200, and 250 ppm (adsorbate) at pH 4.5 was prepared, added to 0.25 mg of the adsorbent composite, stirred at 50 rpm for 90 min at 20 °C, then an aliquot was filtered at 0.25 µm and transferred to an ICP-OES 9820 Shimadzu (Kyoto, Japan).

The equilibrium adsorption capacity of heavy metals (*q_e_*) (mg/g) was determined through Equation (1) [30].

(1) $$q_{e}=\frac{V\left(C_{0}-C_{e}\right)}{m},$$

where *C_e_* is the adsorbate concentration at equilibrium (mg/L); *C*_0_ is the initial concentration of the adsorbate (mg/L); *V* is the volume of the solution (L); *m* is the mass of adsorbent (g).

From the *q_e_* and *C_e_* data, the adsorption isotherm was constructed and adjusted to the Langmuir, Freundlich, and Redlich–Peterson models [30], taking the *R*^2^ value as the convergence criterion and chi-square analysis (Chi-sq).

Langmuir model

(2) $$\frac{C_{e}}{q_{e}}=\frac{1}{q_{m}k_{L}}+\frac{C_{e}}{q_{m}},$$

where *q_e_* is the equilibrium adsorption capacity (mg/g); *k_L_* is the Langmuir constant (L/mg); *C_e_* is the equilibrium adsorbate concentration (mg/L); *q_m_* is the monolayer adsorption capacity (mg/g).

The affinity between adsorbate and adsorbent can be predicted using the Langmuir parameter to form the dimensionless separation factor *R_L_*, which is used to predict whether an adsorption system is “favorable” or “unfavorable” [31].

(3) $$R_{L}=\frac{1}{\left(1+k_{L}C_{0}\right)},$$

where *C*_0_ is the initial concentration of the solution (mg/L).

If: *R_L_* > 1, Unfavorable; *R_L_* = 1, Linear; 0 < *R_L_* < 1, Favorable; *R_L_* = 0, Irreversible.

Freundlich’s model

(4) $$\log q_{e}=\log k_{f}+\frac{1}{n}\log C_{e},$$

where *k_f_* (mg^1−1/*n*^L^1/*n*^/g) and 1/*n* are Freundlich constants.

Redlich–Peterson model

(5) $$q_{e}=\frac{K_{R}C_{e}}{1+a_{R}C_{e}^{g}},$$

where *K_R_* (L/g), *a_R_* (L/mg)*^g^*, and g are the isotherm constants.

### 2.8. Evaluation of Adsorption Kinetics

The kinetics describes the adsorption speed of the adsorbate in the adsorbent and determines the time in which equilibrium is reached [32].

An amount of 250 mL of a multimetal solution of As, Cd, Pb, and Zn of 10 ppm at pH 4.5 was prepared. Of the adsorbent composite, 0.25 mg were added and stirred at 50 rpm at 20 °C, and contact times of 0, 30, 60, 90, 120, and 150 min were used. For each time, an aliquot was filtered at 0.25 µm and transferred to an ICP-OES 9820 Shimadzu (Kyoto, Japan).

With the data obtained, the adsorption kinetics were modeled using pseudo first-order, pseudo second-order, and intraparticle diffusion models [33], taking the *R*^2^ value as the convergence criterion and chi-square analysis (Chi-sq).

Pseudo first-order kinetic model

(6) $$\ln\left(q_{e}-q_{t}\right)=\ln\left(q_{e}\right)-k_{1}t,$$

where *q_e_* is the adsorption capacity of the solute on the adsorbent at equilibrium (mg/g); *q_t_* is the adsorption capacity of the solute on the adsorbent at time *t* (mg/g); *k*_1_ is the kinetic constant of the pseudo first-order model (min^−1^); *t* is the contact time (min).

Pseudo second-order kinetic model

(7) $$\frac{t}{q_{t}}=\frac{1}{k_{2}q_{e}^{2}}+\frac{t}{q_{e}},$$

where *k*_2_ is the kinetic constant of the pseudo second-order model (g/(mg·min)); *t* is the contact time (min).

Kinetic model of intraparticulate diffusion

(8) $$q_{t}=k_{id}t^{1/2}+C,$$

where *k_id_* (mg·min^−1/2^/g) and *C* (mg/g) are the constant intraparticle diffusion rate.

If *C* is equal to zero, the only control step is intraparticle diffusion; if *C_i_* ≠ 0, it indicates that the adsorption process is quite complex and involves more than one diffusive resistance.

## 3. Results and Discussion

### 3.1. Clay Cation Exchange Capacity

The cation exchange capacity (CEC) allows knowing the ability of clay to absorb cations in such a way that they can be easily replaced by competitive ions. It can be considered as the equivalent of the negative charges of clays [34], this being a predictive factor of metal extractability [35].

It was observed that natural clay presented CEC 4.95 ± 0.17 cmol/kg and that after activation it increased significantly (*p*-value < 0.05) up to 42.64 ± 108 cmol/kg. That is, the treatment with NaCl allows the activation of the adsorption sites [36] due to the exchange of the divalent cation Ca^2+^ and Al^3+^ by the monovalent cation Na^1+^ present in clays, thus improving the thixotropic behavior of clay in suspension and dispersion. This exchange can be represented using Equation (9) [37].

(9) $$CaClay+2Na^{1+}\to 2NaClay+Ca^{2+},$$

On the other hand, activation increases the contact surface area, increasing the adsorption/absorption capacity in the interlamellar spaces and producing a high swelling capacity, which enhances the adsorption capacity of heavy metals [18,38,39].

### 3.2. X-ray Analysis of Clay

The X-ray diffraction technique makes it possible to demonstrate the structural and compositional modifications of clay materials subjected to physical or chemical treatments [40,41].

**Figure 1:**
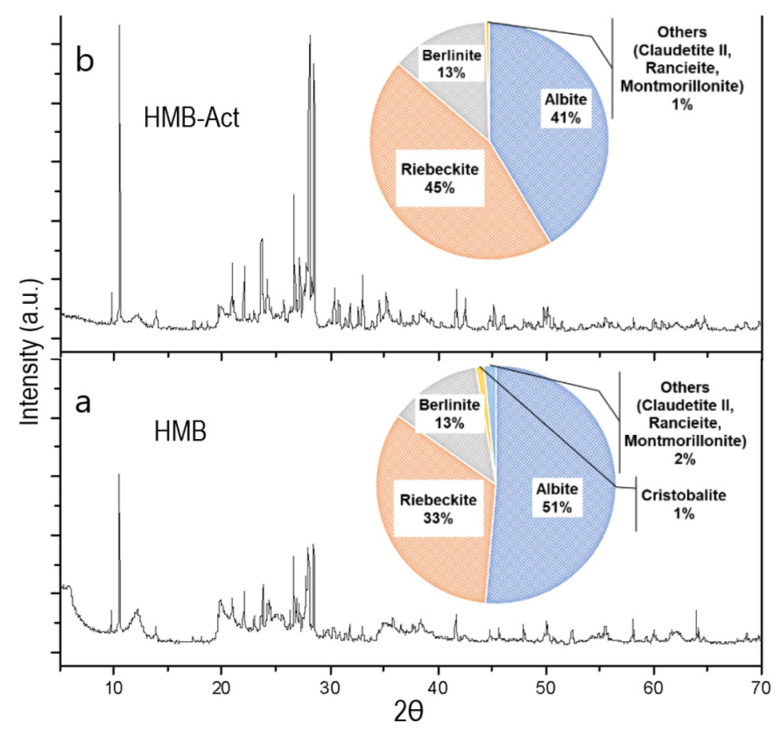
X-ray diffractogram of clay; (**a**) natural clay HMB, (**b**) active clay HMB-Act.

It was observed that the natural clay presents a main reflection of 001 peak at 15.07 Å, which is characteristic of the smectic, presenting a majority composition of Albite (AlNaO_8_Si_3_) (51%), Riebeckite (Al_0.354_Ca_0.014_F_1.253_Fe_4.36_H_0.892_K_0.29_Li_0.344_Mn_0.182_Na_2.024_ O_22.892_Si_7.76_) (33%), Berlinite (AlO_4_P) (13%), Cristobalite (SiO_2_) (1%), and minor composition (<1%), of Claudetite, Montmorillonite, and Rancieite (Figure 1). After activation, it was observed that the peak 001 disappeared, indicating structural modification [41,42]. In this case, the main component is Riebeckite (45%), followed by Albite (41%), confirming the increase of Na^1+^ cations in the activated clay (HMB-Act).

### 3.3. Particle Size, ζ Potential, and SEM Images of the Clay and Hydrocolloid

The particle size of natural HMB clay showed three groups (Table 2), 53.0% corresponded to a mean size of 839.5 nm, 46.4% reported 239.8 nm, and 0.6% 37.6 nm. After activation, it was observed that the size decreases considerably, and 97.4% presented a mean size of 487.5 nm.

This decrease in size is due to the treatment of the clays with phosphoric acid, which dissolves the carbonates, eliminates oxides present in the octahedral structure of the native clay, and destroys the mineral part, generating amorphous silica and active sites that will improve the adsorption centers. On the other hand, the acid action during activation produces dehydroxylation and the elimination of metal cations from octahedral sites produces new pores suitable for adsorption [12,43,44].

Regarding the CH hydrocolloid, it was observed that 95.8% reported a mean size of 421.7 nm, this size would allow the improvement of the electrostatic and chemical interactions during the adsorption process of heavy metals and the formation of colloids and suspension due to its ζ potential (−27.14 mV) (Table 3) [18,45,46,47,48].

The ζ potential allows knowing the solution stability of powdered materials, the absolute value between 21 to 40 mV indicates medium stability, and <20 mV allows easy agglomeration and sedimentation [49,50,51]. In this study, it was observed that activated clay presents a medium stability (39.91 mV, measured at neutral pH) (Table 2).

The reported values of ζ potential for HMB-Act and CH allow the establishment of a good electrostatic attraction due to the greater number of carboxyl, hydroxyl, and carbonyl groups, as evidenced in the IR analysis. These results show that these materials can easily hydrate and interact with metal cations [9,52,53,54]. As for HMB-Act, the activation with NaCl allows the potential to improve, because Na^1+^ ions are extending in a diffuse electric double layer, according to the diffuse double layer theory [9,55,56,57].

**Figure 2:**
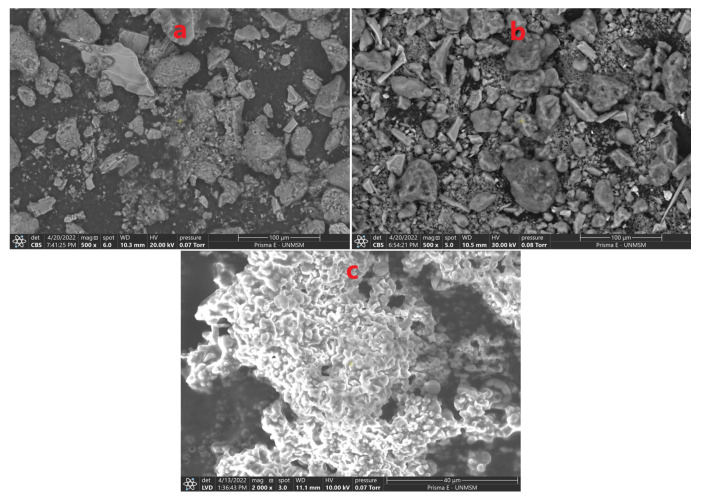
SEM images; (**a**) natural clay HMB, (**b**) activated clay HMB-Act, (**c**) CH hydrocolloid.

The ζ potential is associated with particle size, degree of hydration, chemical nature, surface topography, and charge density on the surface of a material [52,57,58]. High absolute values are an indicator of smaller particle sizes being able to reach nanometric levels, and this was observed in activated clay (Figure 2).

### 3.4. Metal Adsorption

It was observed that the adsorption percentage followed the order HMB-Act/CH > Natural HMB/CH > HMB-Act > Natural HMB (*p*-value < 0.05), that is, the activation of the clay improves the multimetal adsorption and the addition of the hydrocolloid improves it even more (Table 3). On the other hand, it was observed that Pb has a better adsorption affinity, removing 78.35% in natural clay, and up to 99.51% for the HMB-Act/CH composite, followed by As (32.32%), Cd (14.16%), and Zn (10.31%), adsorbing up to 108.14 mg/g of Pb in equilibrium at 120 min.

The Pb affinity is due to the competition by the metal ions in the multimetal system for the active sites of the composite materials. This fact gives rise to an antagonistic effect, which largely depends on the ionic radius, hydration radius, hydration enthalpy, and cation solubility [22,23,59]. Thus, Pb^2+^ (1.20 Å) presents a higher ionic radius, which justifies its greater adsorption [40,60,61,62], followed by Cd (0.97 Å) > Zn (0.74 Å) > As (0.47 Å), substituting the Na^1+^ ions (0.95 Å) of the three-dimensional network of activated clays [63,64]. However, the opposite happens with the hydration radius [59,65], although this also depends on the type of adsorbent material, and the initial multimetal concentration decreases when this is higher, due to the overlapping of active sites [19,60].

This demonstrates the viability of activated natural clay as an effective adsorbent, due to increased contact area, pore-volume, ion exchange capacity, and decreased particle size [16,40,41], presenting specific adsorption at the edges of the nanocomposite structure, caused by the formation of complexes with the hydroxyl and oxygen groups of Si-O [40,66,67].

Materials formulated with activated clays show adsorption levels of around 95% for Pb [40,41,66]. While for activated materials of plant origin, values of around 90% removal are reported [7,17,25,30]; thus, the values found are encouraging for the use of this formulated compound.

### 3.5. IR Analysis of Composites Subjected to Adsorption

**Figure 3:**
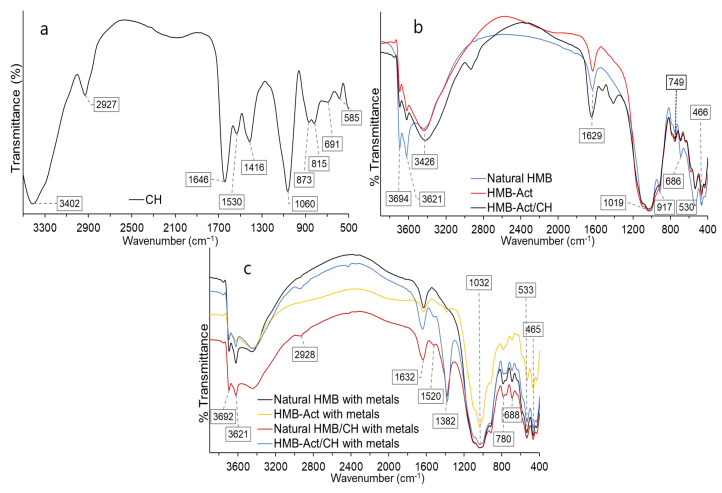
IR spectra; (**a**) hydrocolloid CH; (**b**) natural clay, activated clay, and activated clay/hydrocolloid; (**c**) materials subjected to adsorption.

Regarding the hydrocolloid CH, a peak was observed around 3400 cm^−1^, which corresponds to a vibration of the -OH and -NH bond (Figure 3a), characteristic of amides and carboxylic acids, which would allow the establishment of hydrogen bridge bonds; at 2927 cm^−1^ asymmetric stretching vibrations of the C-H bond are presented, corresponding to the hydrocarbon chains of carbohydrates; another zone with high intensity is found around 1646 cm^−1^, it corresponds to a vibration of the stretching of the carbonyl group -C=O, and -OH stretching of the water present, which suggests high hygroscopicity [68,69].

Around 1530 cm^−1^, low intensity spectra are observed, corresponding to the stretching vibrations of the COO- and -C=O bonds of the carboxylate anions [70]; at 1416 cm^−1^ stretching of the -C-O, -C-H, and -OH single bonds were observed; at 1060 cm^−1^ a high intensity peak is presented, which would be due to the manifestation of bond stretching -C-O, C-O-C, C-OH, a peak at 815 cm^−1^ of low intensity that indicates deformation of the -CH_2_ bond corresponding to methylene groups, while between 800 and 580 cm^−1^ different low intensity spectra are presented, this area is known as the “fingerprint” of the materials, these spectra are attributed to stretching of the -C-H and -C-O bonds, belonging to starches and glucose, which is characteristic of hydrocolloids from algae [13,27,70,71,72,73].

Regarding the natural clay (HMB Natural) and activated (HMB-Act) (Figure 3b), a band at 3694 cm^−1^ corresponds to the stretching -OH of the hydroxyl groups of the internal surface (Si-OH) of the tetrahedral layer, while the band around 3621 cm^−1^ corresponds to -OH stretching the internal hydroxyl groups (Al-OH) of the octahedral layer, confirming the 2:1 arrangement of the smectite. On the other hand, the band around 3426 cm^−1^ and 1629 cm^−1^ is due to -OH stretching vibrations of adsorbed water molecules [74,75].

The shoulder-type band at 1090 cm^−1^ corresponds to Si-O stretch (out of plane), while at 1019 cm^−1^ Si-O stretching (in the plane) is present; at 917 cm^−1^ the natural clay HMB presents a peak and is attributed to the bending vibrations of AlAlOH, AlFeOH, and AlMgOH, causing deformation of hydroxyl groups on the internal surface [76,77]; however, HMB-Act does not present this band, which would confirm the substitution of trivalent and bivalent cations by Na^1+^ [12,77]. While the peaks 749 and 686 cm^−1^ correspond to quartz vibrations present in the clay matrix [26,78].

The band at 530 cm^−1^ and 466 cm^−1^ corresponds to an Al-O-Si deformation and Si-O-Mg and Si-O-Fe vibration bending, respectively [26], presenting higher intensity for HMB-Act, which shows the substitution of trivalent ions (Al^3+^ and Fe^3+^) to monovalent Na^1+^ in the octahedral sheet [12,75].

Regarding the HMB-Act/CH composite (Figure 3b), a peak with high intensity is observed around 2900, 1500, and 1400 cm^−1^, which are attributed to the hydrocolloid CH, and is mainly due to the presence of -OH, -CO, -COO groups, which allows the increase of the active metal adsorption centers [17,20,23].

In materials and composites subjected to adsorption, a considerable decrease in peak intensity was observed around 3400, 2926, 1630, 1430 cm^−1^, and in the fingerprint field, mainly for HMB-Act/CH (Figure 3c). This would be due to the functional groups of the clay and hydrocolloid that would be responsible for the complexation of Pb^2+^ and Cd^2+^ and Zn^2+^, due to the O and N binding atoms [15,79,80]. Likewise, this would be due to the synergistic effect of the functional groups -OH of Al-OH and Si-OH of the octahedral sheet in the activated clay and -NH and -OH of the hydrocolloid [40,41,80,81,82].

On the other hand, the appearance of a 1380 cm^−1^ peak with high intensity was observed (Figure 3c), which clearly indicates the complexation of heavy metals, mainly Pb [17,80].

In this sense, it can be considered that the formulated composite (HMB-Act/CH) has a high capacity for heavy metal removal, evidencing the synergy of the functional groups of each material, so inorganic materials at the nanoparticulate level and biological materials, such as the *Nostoc Sphearicum* algae, present good ability to complex monovalent, bivalent, and trivalent heavy metals [17,24,30,41,42,80,83,84,85,86].

### 3.6. Metal Adsorption Kinetics in the Composite

The study of adsorption kinetics is important in the design of adsorption systems, allowing residence times, reaction rates, and reactor sizing being established [24,87].

The pseudo first-order model describes the adsorption of liquid–solid phase systems as a function of adsorption capacity, considering that the binding site occupancy rate is proportional to the number of unoccupied sites on the sorbent [14,88].

The pseudo second-order model considers the adsorption capacity of solid phases, due to the chemical bonds (chemisorption with strong interactions) in the adsorbent monolayer. It also describes the occupancy rate of the adsorption sites, proportional to the square of the number of unoccupied sites in the sorbent [24,88]. The intraparticle diffusion model considers the probability that the adsorbate is transported from a concentrated zone to the adsorbent through diffusion, this being the stage that limits the speed in many adsorption processes, generally for discontinuous agitation processes, where the adsorption varies almost proportionally to t^1/2^ at the point of contact time t [88].

The pseudo first-order model showed values of *R*^2^ > 0.944, and the pseudo second-order model reported *R*^2^ > 0.980 (Table 4). The fact that the fit of the pseudo first-order model is slightly lower could be attributed to the limitations of the boundary layers that control the physical adsorption processes [21,22]. Therefore, the adsorption of metals would be subject to chemisorption processes, which allows a better description of the pseudo second-order model [16,23].

**Figure 4:**
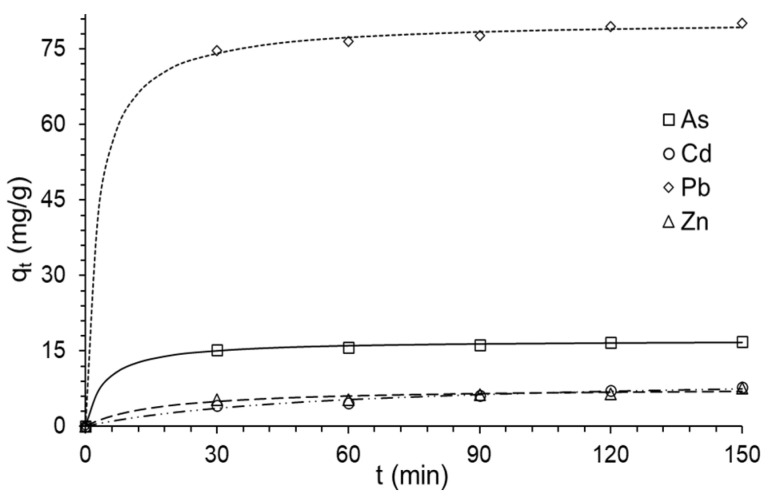
Second-order kinetics modeling.

It was observed that adsorption takes place rapidly during the first 30 min (Figure 4), due to the availability of the active sites on the surface of the composite, establishing the complexation of the -NH, -C=O, -OH, Al-O-Si, and Si-O groups of the clay and hydrocolloid (chemisorption process); for longer times, adsorption is very slow, with intraporous adsorption occurring mainly from 90 min onwards. The increase of adsorption is not significant, this behavior is usual for clay materials subjected to Pb, Cd, and Zn adsorption [16,80]. The adsorption equilibrium is influenced by the nature of the adsorbent and adsorbate, mainly by the functional groups of the active sites, particle size, ion exchange capacity, and ζ potential [16,21].

The kinetic parameters of the pseudo second-order model (Table 4) reported that q_e_ (equilibrium adsorption capacity) is higher for Pb, followed by As, Zn, and Cd; demonstrating greater specificity of the composite for Pb, which is characteristic for clay adsorbents [19,21,23,62,80,85,89,90].

The adsorption rate, *k*_2_, ranged from 0.002 to 0.025 g/mg·min. These values depend on the nature of the adsorbent and adsorbate and the medium conditions. *q_e_* does not show any behavior, although low values suggest lower adsorption capacity in multimetal systems when treated with inorganic and organic adsorbents [14,21,80,84,89,91].

Adsorption processes can be explained through four processes: (*i*) surface migration, (*ii*) film diffusion, (*iii*) intraparticle or pore diffusion, and (*iv*) sorption at interior sites. Stages *i* and *iii* occur spontaneously due to the availability of metals near the surface, whereas stages *ii* and *iv* generally control the rate of adsorption, and the intraparticle model makes it possible to explain this phenomenon [19,89].

The intraparticular model (*R*^2^ > 0.70) showed that the *k_id_* intraparticulate rate constant is higher for Pb, followed by As > Zn > Cd (Table 4), suggesting that surface migration and intraparticulate diffusion occur rapidly, especially for Pb and As. While low *k_id_* values would be subject to diffusion in the film and sorption in interior sites of the adsorbent, and the higher hydration radius of Zn and Cd [65], would be the conditioning factors of the adsorption rate [89].

On the other hand, the constant *C*, related to the thickness of the boundary layer, reported higher values for Pb and As (Table 4). If *C* is equal to zero, the only control step is intraparticle diffusion; if *C_i_* ≠ 0, it indicates that the adsorption process is quite complex and involves more than one diffusive resistance [41,90,92], confirming the information reported by *k_id_*, which is that the metals diffuse slowly in the pores of the HMB-Act/CH composite, which constitutes a limiting step [40,89,90].

### 3.7. Metal Adsorption Isotherms in the Composite

The interaction behavior of adsorbate and adsorbent at equilibrium is described through adsorption isotherms [10,24].

The Langmuir equation is applicable to homogeneous sorption, each molecule has the same sorption activation energy and is based on the assumptions that (*i*) adsorption can only occur at a fixed number of defined localized sites, (*ii*) each site may contain only one adsorbate molecule (monolayer) at all sites, and (*iii*) there is no interaction between adsorbed molecules even at adjacent sites [10,31,93].

**Figure 5:**
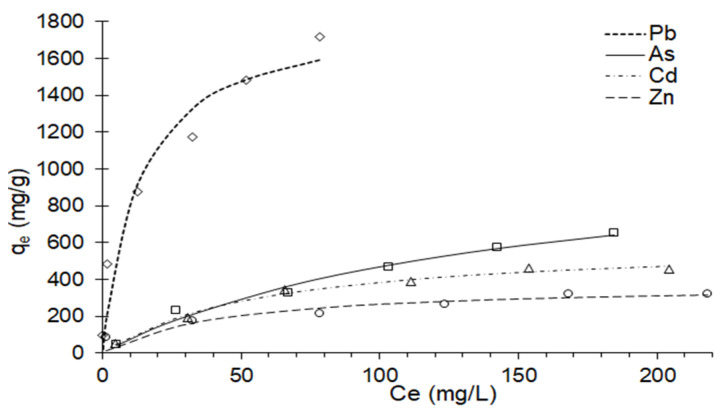
Isotherms fitted to the Langmuir model.

Regarding the Langmuir isotherm (*R*^2^ > 0.92 and chi-sq < 51.99), many multimetal sorption systems have been represented by this model. The *q_max_* value, which represents the maximum adsorption capacity at the monolayer level [31] for the nanocomposite, showed selectivity in the order Pb > As > Cd > Zn (Figure 5), being usual behavior for clays, although the values obtained are higher than those reported elsewhere [63,85,89,94]. This would be due to the activation of the clay, nanoparticulate size, and the ζ potential that the clay and the hydrocolloid present, giving them stability in suspension, which allows them to be in greater contact with the multimetal solution.

The *K_L_* parameter showed a higher value for Pb (Table 5), which indicates that the nanocomposite shows a high affinity for this metal due to the assumption of a finite number of identical active sites in the nanocomposite. While As, Cd, and Zn show similar values, this is related to the similar ionic radius that they present. This behavior is characteristic for clays [23,63,64,85,89,94].

The separation constant *R_L_* indicates whether the adsorption system is favorable or unfavorable, when *R_L_* > 1, unfavorable; *R_L_* = 1, linear; 0 < *R_L_* < 1, favorable; *R_L_* = 0, irreversible [10,31,95]. The results found showed favorable adsorption for metals (Table 6); however, the behavior for Pb at the initial concentrations under study was more favorable, although at the initial concentration of 10 mg/L for the metals As, Cd, and Zn, they tended to be unfavorable due to antagonism with Pb, which has a greater preference for the active sites of the monolayer, caused by a greater ionic radius, which allows it to substitute the Na^1+^ ions of tetrahedral structure of the nanoclay and the functional groups of the hydrocolloid in the nanocomposite [10,31,96].

The Freundlich isotherm, which proposes multilayer sorption with a heterogeneous energetic and surface distribution on active sites and/or interactions between sorbed species can be used to describe heterogeneous and multicomponent systems [63,97,98].

The Freundlich model (*R*^2^ > 0. 95 and Chi-sq < 41.71) (Table 5) reported values of the relative adsorption capacity K_F_ in the order Pb > Zn > Cd > As. The nanocomposite shows a relative preference for Pb, which should not be confused with the percentage of removal since adsorption is relative to agitation conditions, pH, temperature, particle size, and hydration radius [22,23,59,63,94,97,99].

The 1*/n* parameter describes the nature of the process, with 1*/n* < 1 indicating higher saturation, measured as the potential availability of different sorption sites on the nanocomposite surface for the adsorbed metals [33,63,100]. If 0 < 1*/n* < 1, it indicates a heterogeneous surface structure with an exponential distribution of the active sites [41]. According to the reported values (Table 5), it was observed that the nanocomposite presented a heterogeneous surface, which is due to the surface characteristics of the nanoclay and the atomized nostoc, giving it high saturation for the adsorption of metals. Although a scale of the values of 1*/n* has not been reported, it could indicate a rapid saturation of Zn in the available active sites, followed by Pb; however, Zn presented a lower percentage of removal, and this could be attributed to its atomic radius [60,61].

On the other hand, values of *n* between 1 and 10 represent favorable absorption [10,23,31,33,40,80,89]. In addition, it was observed that *n* reported values that are in the range of 1.64 to 3.35, which suggests favorable adsorption in the nanocomposite.

The Redlich–Peterson empirical model, called the “three-parameter equation”, which makes it possible to represent adsorption equilibria over a wide concentration range [31,63,97], reported *R*^2^ values between 0.83 and 0.99, suggesting a good fit, and would allow criteria to be taken in reactor designs based on the initial concentrations of multimetal mixtures. Although the parameters of this model lack a physicochemical and thermodynamic explanation; a correlation of the parameter *K_R_* and g with the parameters of the Langmuir and Freundlich model, percentage of removal, ionic radius, and functional groups (IR analysis) has not been observed, although the *a_R_* parameter shows a correlation with the percentage of metal removal. 

## 4. Conclusions

The characteristics of the nanocomposite showed the synergistic behavior of the components, active nanoclay (HMB-Act) and hydrocolloid (CH), evidenced through IR, CEC, X-ray, particle size, and ζ potential analysis, with better qualities for the removal of metals in multimetal systems in the order Pb(99.52%) > As(33.12%) > Cd(16.91%) > Zn(13.07%) for an initial solution of 10 ppm multimetal solution, 100 mg of compound per liter of solution, and pH 4.5. The pseudo first- and pseudo second-order kinetics were adjusted to the adsorption kinetics. The Freundlich and Langmuir models for the adsorption isotherms fit well with *R*^2^ values around 0.98. The nanocomposite is a material with high potential as a heavy metal removal agent in wastewater.

## Figures and Tables

**Table 1:** Clay collection coordinates.

Community	District	Region	Coordinates		Altitude (m)	Collected Period
			S	W		
Huancabamba	José María Arguedas	Apurímac	13°43′58″	73°20′38″	3682	April/2021

**Table 2:** Particle size and ζ potential.

Material	NICOMP Distribution			Gaussian Distribution			ζ Potential (mV)
	Peak	Size (nm)	%	$\bar{x}$	SD	CV (%)	
CH	1	43.4	4.2	454.0	269.2	59.3	−27.14
	2	421.7	95.8				
HMB	1	37.6	0.6	372.7	200.2	53.7	−19.31
	2	239.8	46.4				
	3	839.5	53				
HMB-Act	1	65	2.6	686.4	505.2	73.6	−39.91
	2	487.5	97.4				

Where: $\bar{x}$, arithmetic mean; SD, standard deviation; CV, coefficient of variability.

**Table 3:** Metals adsorption (%).

Clay or Composite	As (WL 197.262 nm)			Cd (WL 326.106 nm)			Pb (WL 405.783 nm)			Zn (WL 213.856 nm)		
	*q_e_* (mg/g)	$\bar{x}$	CV (%)	*q_e_* (mg/g)	$\bar{x}$	CV (%)	*q_e_* (mg/g)	$\bar{x}$	CV (%)	*q_e_* (mg/g)	$\bar{x}$	CV (%)
Natural HMB	18.97	22.25 ± 0.24a *	1.10	2.40	3.88 ± 0.09a	2.41	85.14	78.35 ± 0.10a	0.13	3.67	5.23 ± 0.30a	5.68
HMB-Act	21.23	24.91 ± 0.51b	2.05	4.17	6.73 ± 0.16b	2.40	88.49	81.43 ± 0.06b	0.07	5.23	7.46 ± 0.51b	6.89
Natural HMB/CH	17.87	30.61 ± 0.45c	1.48	6.07	12.21 ± 0.60c	4.95	107.13	98.59 ± 0.44c	0.47	5.40	8.61 ± 0.33c	3.85
HMB-Act/CH	18.87	32.32 ± 0.51d	1.59	7.03	14.16 ± 0.42d	2.96	108.14	99.51 ± 0.53d	0.54	6.47	10.31 ± 0.40d	3.89

Where Natural HMB is natural HMB clay subjected to adsorption; HMB-Act is activated HMB clay subjected to adsorption; Natural HMB/CH is composite subjected to adsorption (natural clay/Hydrocolloid); HMB-Act/CH is a composite subjected to adsorption (activated clay/Hydrocolloid); WL, Wavelength. $\bar{x}$, arithmetic mean; SD, standard deviation; CV, coefficient of variability. * Different letters indicate a significant difference, evaluated with the Tukey test at 5% significance.

**Table 4:** Parameters of the kinetic models for the HMB-Act/CH composite.

Metal Ion	Pseudo First-Order				Pseudo Second-Order				Intraparticle Diffusion			
	*q_e_*	*K*_1_	*R* ^2^	Chi-sq	*q_e_*	*k*_2_	*R* ^2^	Chi-sq	*k_id_*	*C*	*R* ^2^	Chi-sq
As	16.57	0.10	0.99	0.04	16.99	0.03	1.00	0.01	1.32	3.58	0.76	2.81
Cd	7.63	0.02	0.97	0.27	9.85	0.00	0.98	0.17	0.60	0.21	0.85	3.82
Pb	78.545	0.0989	0.99	0.09	80.76	0.00	0.99	0.03	6.260	16.841	0.77	15.15
Zn	5.98	0.0345	0.94	0.28	7.00	0.01	0.99	0.17	0.508	0.571	0.93	0.57

**Table 5:** Adsorption isotherm parameters for the composite.

Metal Ion	Langmuir Isotherm				Freundlich Isotherm					Redlich–Peterson Isotherm				
	*q_max_*	*K_L_*	*R* ^2^	Chi-sq	*K_F_*	1*/n*	*n*	*R* ^2^	Chi-sq	*K_R_*	*a_R_*	*g*	*R* ^2^	Chi-sq
As	1117.78	0.01	0.98	21.43	27.55	0.61	1.64	0.99	16.00	0.58	−1.02	−0.04	0.96	11.95
Pb	1855.51	0.08	0.94	51.99	332.39	0.38	2.66	0.99	41.71	14.65	−2.28	−0.41	0.83	18.24
Cd	606.08	0.02	0.99	7.82	44.95	0.45	2.22	0.95	26.81	8.13	0.00	1.24	0.99	12.81
Zn	374.13	0.02	0.92	57.42	64.11	0.30	3.35	0.96	9.73	415.13	6.18	0.71	0.96	14.43

**Table 6:** RL values for adsorption evaluated through the Langmuir model.

Initial Concentration, C_0_ (mg/L)	As		Pb		Cd		Zn	
	Final Concentration, C_f_ (mg/L)	*R_L_*	Final Concentration, C_f_ (mg/L)	*R_L_*	Final Concentration, C_f_ (mg/L)	*R_L_*	Final Concentration, C_f_ (mg/L)	*R_L_*
10.0	5.15	0.93	0.17	0.56	4.52	0.85	1.23	0.81
50.0	26.78	0.73	1.87	0.20	31.04	0.54	32.63	0.46
100.0	67.21	0.57	12.53	0.11	65.89	0.37	78.56	0.30
150.0	103.35	0.47	32.50	0.08	111.11	0.28	123.56	0.22
200.0	142.52	0.40	51.87	0.06	153.97	0.23	168.12	0.18
250.0	184.55	0.35	78.40	0.05	204.47	0.20	218.08	0.15

## Data Availability

The data presented in this study are available in this article.
